# Sulfur compounds navigate redox processes, leukotriene synthesis, and ω-hydroxylation of leukotriene B4 in neutrophil interaction with the bacteria *Salmonella typhimurium*: the way to manipulate neutrophil swarming

**DOI:** 10.3389/fimmu.2025.1606408

**Published:** 2025-10-15

**Authors:** Ekaterina A. Golenkina, Sofia V. Navarnova, Galina M. Viryasova, Svetlana I. Galkina, Tatjana V. Gaponova, Yulia M. Romanova, Galina F. Sud’ina

**Affiliations:** ^1^ Belozersky Institute of Physico-Chemical Biology, Lomonosov Moscow State University, Moscow, Russia; ^2^ Faculty of Bioengineering and Bioinformatics, Lomonosov Moscow State University, Moscow, Russia; ^3^ National Research Center for Hematology, Russia Federation Ministry of Public Health, Moscow, Russia; ^4^ Gamaleya National Research Centre of Epidemiology and Microbiology, Moscow, Russia

**Keywords:** neutrophil, *Salmonella typhimurium*, leukotriene B4, reactive oxygen species, glutathione, neutrophil swarming

## Abstract

Neutrophils are the first immune cells recruited by invading pathogens. During interaction with bacteria, neutrophils synthesize leukotriene B4, a potent chemoattractant that, in conjunction with the primary bacterial chemoattractant *N*-formyl-l-methionyl-l-leucyl-l-phenylalanine (fMLP), stimulates the formation of neutrophil clusters surrounding pathogens. Hydrogen sulfide (H_2_S) plays a critical role in the regulation of host–bacteria interactions, and bacteria are known to use H_2_S in response to host-induced oxidative stress. The purpose of this study was to investigate the regulatory role of H_2_S in neutrophil cellular responses in an experimental model of neutrophil interaction with *Salmonella typhimurium*. The application of H_2_S donor (sodium hydrosulfide hydrate, NaSH) during the interaction of neutrophils with bacteria increased the leukotriene synthesis stimulated by the peptide fMLP. NaSH significantly suppressed the reactive oxygen species (ROS) formation in neutrophils. When phorbol-12-myristate-13-acetate (PMA) was used in cell pretreatment before the addition of fMLP, a decreased leukotriene synthesis and an increased ROS formation in cells were observed. Not producing ROS disulfide stress induced by diamide, in combination with NaSH, synergistically increased the fMLP-induced leukotriene synthesis during the interaction of neutrophils with the bacteria *S. typhimurium*. The data obtained demonstrate that not producing ROS disulfide stress increases leukotriene synthesis in the presence of H_2_S-producing compounds.

## 1 Introduction

The innate immune system regularly scans our bodies for pathogens and tissue damage (1). Human polymorphonuclear leukocytes (neutrophils, PMNLs), designed to engulf and destroy invading pathogens, are the first line of host defense (2). Neutrophils involved in the inflammatory process synthesize leukotrienes. Leukotriene B4 (LTB_4_) increases the killing of bacteria (3). Leukotriene synthesis in inflammatory loci is important for the cooperative recruitment of neutrophils to the site of microbial invasion, so-called neutrophil swarming (4, 5).

What factors influence the synthesis of leukotrienes in the inflammation foci? The catalytic cycle of 5-lipoxygenase (5-LOX) involves peroxy derivatives of polyunsaturated fatty acids; therefore, the synthesis of leukotrienes is very sensitive to the redox status of the cell. In intact cells, the formation of leukotrienes requires the presence of a certain threshold concentration of fatty acid hydroperoxides (6, 7).

Neutrophils kill *Salmonella* bacteria by generating overwhelming oxidative stress through NADPH oxidase and myeloperoxidase (8). Non-typhoidal *Salmonella* responding to oxidative stress produces hydrogen sulfide (H_2_S) (9). Mammalian cells synthesize H_2_S from sulfur-containing amino acids, but mainly are exposed to H_2_S from exogenous sources of this signaling molecule, particularly from gut microbes. H_2_S increases glutathione (GSH) biosynthesis (10) and influences energy metabolism (11), and it activates several antioxidant mechanisms, including NADPH oxidase enzyme inhibition (12). H_2_S is an efficient scavenger of reactive oxygen species (ROS), along with GSH-level supporting activity (13). During inflammation, the oxidation status of cellular protein thiols changes dynamically, with reversible thiol–disulfide exchange between protein thiols and the intracellular pool GSH/glutathione disulfide (GSSG).

Activated PMNLs release ROS when killing bacteria, and the activity of many proteins under oxidative stress is modulated by the oxidation of thiol groups. Oxidative stress caused by hydrogen peroxide is associated with the formation of non-native disulfide bonds in thiol-containing compounds, so-called disulfide stress (14).

Diamide reacts quickly with intracellular thiols, not producing ROS. Diamide mediated a strong increase in reversibly oxidized thiols in various metabolic enzymes by forming disulfides between thiols (15). In this study, diamide was used to trigger disulfide stress. This is a suitable approach for the non-oxidant alteration of cellular thiols to separate the forming disulfide from other ROS-induced oxidation processes (16).

Dynamic changes of the intracellular pool GSH/GSSG upon diamide and sodium hydrosulfide hydrate (NaSH) exposure can mimic the interference of the oxidant and antioxidant mechanisms in neutrophil–bacteria interaction. We propose that the entry into the cell of H_2_S, which suppresses the microbicidal activity of neutrophils, will provoke neutrophils to induce leukotriene synthesis for attracting more neutrophils to microbe invasion. It was found that disulfide stress in combination with the GSH-supporting compound NaSH synergistically supports leukotriene synthesis during the interaction of neutrophils with the bacteria *Salmonella typhimurium*.

## 2 Materials and methods

### 2.1 Materials

Dulbecco’s phosphate-buffered saline (D-PBS) with magnesium but without calcium, Hank’s balanced salt solution with calcium and magnesium but without phenol red and sodium hydrogen carbonate (HBSS), NaSH, diamide, *N*-formyl-l-methionyl-l-leucyl-l-phenylalanine (fMLP), 6-aminonicotinamide (6-AN), and fibrinogen from human plasma were purchased from Sigma (Steinheim, Germany). Dextran T-500 was from Pharmacosmos (Holbæk, Denmark). The acetoxymethyl ester (AM)-conjugated carboxy-2',7'-dichlorodihydrofluorescein diacetate (H_2_DCF-DA), fura-2 AM, and the goat anti-mouse IgG secondary antibody, Alexa Fluor™ 488, were purchased from Thermo Fisher Scientific (Waltham, MA, USA). Purified mouse anti-5-LOX monoclonal antibodies (mAbs) were from BD Biosciences (Franklin Lakes, NJ, USA). The GSH/GSSG-Glo™ and RealTime-Glo™ Extracellular ATP Assay kits were from Promega Corp. (Madison, WI, USA).

Bacteria (*S. typhimurium* strain IE 147) were obtained from the collection of the N.F. Gamaleya National Research Center for Epidemiology and Microbiology (Moscow, Russia). Bacteria were grown in Luria–Bertani broth to a concentration of 1 × 10^9^ colony-forming units (CFU) per milliliter. In this study, non-opsonized bacteria were used.

### 2.2 Isolation of PMNLs

Human PMNLs were isolated from freshly collected citrate-anticoagulated blood obtained from healthy adult volunteers of both sexes. Leukocyte-rich plasma was obtained from donated blood by sedimentation in the presence of dextran T-500. Granulocytes were obtained as described (17). Cell viability was examined using the trypan blue exclusion method. PMNLs (96%–97% purity and 98%–99% viability) were stored at room temperature in calcium-free D-PBS containing 1 mg/ml glucose until use.

### 2.3 Study of 5-LOX product synthesis in cells

PMNLs [(1.3–1.6) × 10^7^/6 ml HBSS with 10 mM HEPES] were placed at 37°C in a CO_2_ incubator for 10 min, and then bacteria or reagents were added. Intact or heat-inactivated *S. typhimurium* bacteria were used. Heat inactivation was performed by incubation in a water bath at 70°C for 1 h (18). *S. typhimurium* and the indicated reagents were added for 30 min, followed by exposure to 0.1 μM fMLP for 10 min. The incubations were stopped by adding an equal volume of methanol (−18°C) with 90 ng PGB2 as an internal standard. The major 5-LOX metabolites—5*S*,12*R*-dihydroxy-6,14-*cis*-8,10-*trans*-eicosatetraenoic acid (LTB_4_), iso-LTB_4_ (5*S*,12*SR*-all-*trans*-diHETE), ω-OH-LTB_4_, ω-COOH-LTB_4_, and 5*S*-hydroxy-6-*trans*-8,11,14-*cis*-eicosatetraenoic acid (5-HETE)—were identified as previously described (19).

### 2.4 Assessment of 5-LOX subcellular localization

PMNLs (10^6^/ml HBSS/HEPES) were incubated in fibrinogen-coated confocal dishes at 37°C with 5% CO_2_ according to the experimental design. After the incubation period, the cells were fixed with 2.5% paraformaldehyde solution followed by acetone permeabilization. The samples were then incubated overnight at 4°C with mouse anti-5-LOX mAb [1:100 in 1% bovine serum albumin (BSA)/PBS]. After washing three times, the samples were stained with the goat anti-mouse Alexa Fluor™ 488 (1:100 in 1% BSA/PBS) for 6 h at 4°C. The cell nuclei were stained with 0.5 μg/ml Hoechst 33342 (Thermo Fisher Scientific, Waltham, MA, USA). Image acquisition was performed using a fluorescence microscope, Olympus IX 83 (Tokyo, Japan), equipped with ×60 oil immersion objective. At least eight random pictures were captured for each sample.

### 2.5 Intracellular ROS assessment

Intracellular ROS accumulation was quantified by measuring the green fluorescence of 2',7'-dichlorofluorescein (DCF). Loading was performed according to the manufacturer’s instruction. Briefly, the neutrophils were incubated in D-PBS supplemented with 5 mM H_2_DCF-DA for 60 min at room temperature followed by washing with PBS, suspended in D-PBS, and then stored at room temperature in the dark until use. Before the experimental treatment, the cells were equilibrated for 5 min in HBSS/HEPES in fibrinogen-coated wells of a 96-well plate (4 × 10^5^ cells/well) at 37°C and 5% CO_2_. The fluorescence intensity at excitation and emission wavelengths of 488 and 525 nm, respectively, was measured using a CLARIOstar multi-mode microplate reader (BMG Labtech, Cary, NC, USA). MARS data analysis software package from BMG Labtech was used to process the data obtained.

### 2.6 GSH/GSSG ratio assessment

Quantitative assessment of the ratio of reduced to oxidized GSH was performed using a commercial luminescence-based system, GSH/GSSG-Glo™ assay. Briefly, two sets of PMNLs (one for total GSH and another for GSSG measurement) in HBSS/HEPES were treated according to the experimental protocol in a white 96-well plate (10^5^ cells/well). The cell lysis and all subsequent manipulations were carried out in strict accordance with the manufacturer’s instructions. Luminescence measurements were made using a CLARIOstar microplate reader. To convert the luminescence intensity values (relative light units, RLU) into GSH and GSSG concentrations, a GSH standard curve (0–16 µM) was used.

### 2.7 Calcium flux assay

Changes in the intracellular calcium concentration ([Ca^2+^]_i_ were detected with ratiometric calcium-sensitive fluorescent dye fura-2 AM. All procedures were performed according to the manufacturer’s instructions, with minor modifications. Briefly, isolated PMNLs (10^7^ cells/ml) were incubated with 1 µM fura-2 AM in D-PBS for 30 min at 37°C. Loaded cells were washed once with PBS and resuspended in D-PBS. Immediately before the experimental procedure, labeled cells were seeded in fibrinogen-coated black 96-well F-bottom plates containing warmed HBSS/HEPES medium, equilibrated for 5 min, and treated according to the experimental design at 37°C in 5% CO_2_. Stimuli were added using reagent injectors integrated into the reader platform. Changes in the fluorescence emitted at 510 nm were measured when excited at both 380 nm (for Ca^2+^-free dye) and 335 nm (for Ca^2+^-bound dye) every 0.6 s. Manipulations were performed on a CLARIOstar microplate reader. MARS data analysis software package was used to process the data obtained. [Ca^2+^]_i_ shifts were assessed based on the changes in the ratio of fluorescence intensities produced by excitation at two wavelengths. Data were quantified using areas under the kinetic curves (AUCs) above the baseline.

### 2.8 ATP assessment

An ATP detection component from the RealTime-Glo™ Extracellular ATP Assay kit was used. In accordance with the manufacturer’s protocol, the lyophilized enzyme/substrate mixture (ATP assay substrate) was reconstituted by HBSS/HEPES to obtain the ATP detection reagent. Just before the experiment, the PMNLs were seeded in fibrinogen-coated solid white 384-well F-bottom plates (5 × 10^4^ cells/well) and pre-incubated for 5 min at 37°C with 5% CO_2_. *S. typhimurium* alone or in combination with NaSH and/or diamide was added for 20 min, followed by treatment with fMLP. The PMNLs incubated without the addition of stimuli were used for data normalization. For total ATP assessment, digitonin (40 µg/ml final concentration) and the ATP detection reagent were added either immediately before or 3 min after the addition of fMLP. After 3 min orbital shaking, the luminescence intensity was measured on a CLARIOstar microplate reader (BMG Labtech, Ortenberg, Germany). MARS data analysis software package from BMG Labtech was used to process the data obtained.

### 2.9 Statistics

Graphing and statistical analysis were performed using GraphPad Prism software version 10.3.1 for Windows. Results are presented as the mean ± SEM. Differences with a *p*-value <0.05 were considered statistically significant. Two-way analysis of variance (ANOVA) followed by Tukey’s multiple comparisons test was used to quantify the 5-LOX product synthesis and ROS assessment. Repeated measures (RM) one-way ANOVA and Tukey’s multiple comparisons test were used to quantify the GSH/GSSG ratio and the calcium flux and total ATP, respectively.

## 3 Results

Leukotriene synthesis induced by fMLP during the interaction of neutrophils with the bacteria *S. typhimurium* was much higher than that induced by fMLP without bacteria (19). Microscopy of the samples sequentially treated with bacteria and fMLP revealed a tendency for the formation of individual neutrophil clusters. It is likely that, under conditions of a high bacterial load, it is precisely the release of LTB_4_ that plays a central role in the qualitative transition of the defense strategy from individual to collective (**Supplementary Figure S1**). We asked how important bacterial cellular metabolism is in the regulation of leukotriene synthesis in neutrophils and compared the leukotriene synthesis during incubation with live bacteria and heat-inactivated bacteria. We found a significant reduction in the effect of bacteria on leukotriene synthesis when heat-inactivated bacteria were used (**Supplementary Figure S2**).

### 3.1 Thiol and ROS-mediated signaling in leukotriene synthesis during neutrophil interaction with *Salmonella* bacteria

**Figure 1:**
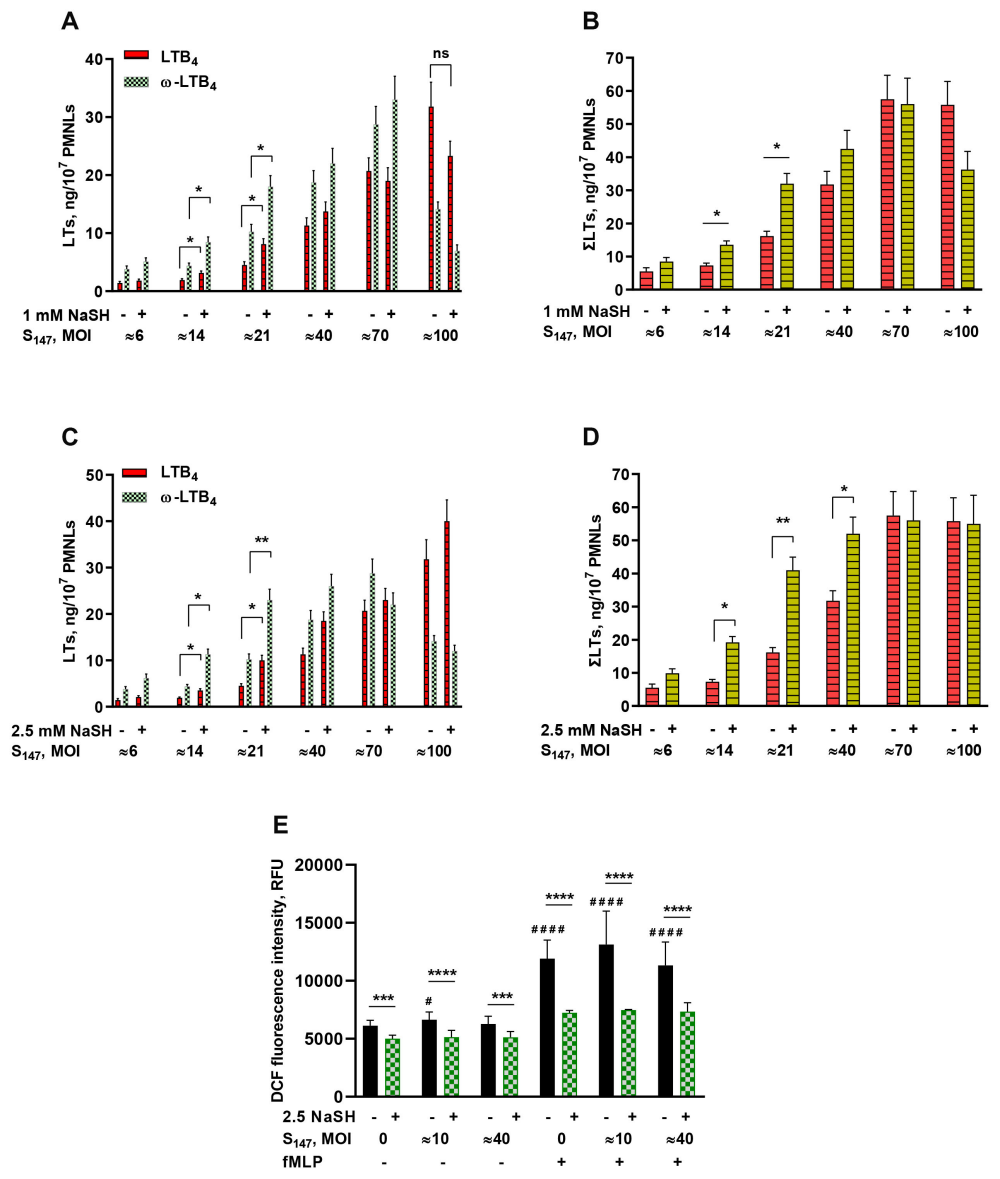
**(A–D)** Effect of sodium hydrosulfide hydrate (NaSH) on leukotriene synthesis in human neutrophils at various bacterial loads. Polymorphonuclear leukocytes (PMNLs) [(1.3–1.6) × 10^7^/6 ml] were pre-incubated for 10 min at 37°C, 5% CO_2_. After 10 min pre-incubation, the PMNLs were exposed for 30 min to *Salmonella typhimurium* (S_147_) bacteria alone or in combination with 1 mM **(A, B)** or 2.5 mM **(C, D)** NaSH, as indicated on the *X*-axis, followed by the addition of *N*-formyl-l-methionyl-l-leucyl-l-phenylalanine (fMLP, 0.1 µM) for 10 min. The bacteria-to-PMNL ratios (multiplicity of infection, MOI) are indicated. After termination of the incubation, the 5-lipoxygenase (5-LOX) products were analyzed. Presented are the absolute values of LTB_4_ and ω-OH-LTB_4_ **(A, C)** and the sum of leukotrienes (LTs) (ΣLTs) **(B, D)** in nanograms per 10^7^ PMNLs. Values shown are the mean ± SEM of three independent experiments performed in duplicate. *p < 0.05, **p < 0.01 [for pairs of data as shown using two-way ANOVA with Tukey’s multiple comparisons test **(A, C)** or by one-way ANOVA **(B, D)**. **(E)** Effect of NaSH on the intracellular reactive oxygen species (ROS) accumulation in neutrophils. After 10 min pre-incubation, PMNLs loaded with H_2_DCFDA were exposed for 20 min to S_147_ bacteria alone (except with MOI = 0) (*black*) or in combination with 2.5 mM NaSH (*green*), as indicated on the *X*-axis. Subsequently, 0.1 µM fMLP was added (indicated) for 30 min, followed by fluorescence detection. Values shown are the mean ± SEM of 2',7'-dichlorofluorescein (DCF) fluorescence intensity measured in three independent experiments performed in triplicate. ^#^ *p* < 0.05, ^####^ *p* < 0.0001 (compared with the corresponding control values); ***p < 0.001, ****p < 0.0001 (for pairs of data indicated as shown by two-way ANOVA with Tukey’s or Sidak’s multiple comparisons test).

Gut microbes may synthesize H_2_S (20). Bacteria use H_2_S in response to host-induced stress factors such as oxidative stress (21). The sulfur compound H_2_S is a highly reactive molecule that can suppress the accumulation of both ROS and reactive nitrogen species (RNS) in inflammatory conditions (22). In the experimental model of neutrophil interaction with the bacteria *S. typhimurium*, the exogenous H_2_S donor NaSH enhanced leukotriene synthesis depending on the bacterial load (**Figure 1**). The bacterial load is represented by the multiplicity of infection (MOI) value, i.e., the ratio between the number of bacteria and PMNLs. The effect was observed after 30 min pre-incubation of the neutrophils and bacteria with NaSH, followed by the addition of 0.1 µM fMLP. The effect was significant at medium values of MOI (**Figures 1A–D**).

NaSH had a moderate antioxidant effect, suppressing the intracellular ROS accumulation in neutrophils including in cells interacting with bacteria and/or stimulated by formyl peptide (**Figure 1E**).

What is the interplay between hydrogen sulfide and oxidative stress?

Oxidative stress is an imbalanced condition caused by the excess production of ROS and the lack of antioxidants (23). Generation of ROS by the catalytically active NADPH oxidase complex is one of the main mechanisms of pathogen degradation by neutrophils (24). Bacteria use various strategies to evade restriction by human neutrophils. Intracellular *Salmonella*, under oxidative stress, initiates the synthesis of the antioxidant H_2_S (9).

H_2_S can scavenge ROS and increase the GSH level in cells (25). In addition, viable bacteria may inhibit the assembly of the phagocyte NADPH oxidase complex, thus preventing synthesis of the microbicide ROS in neutrophils.

**Figure 2:**
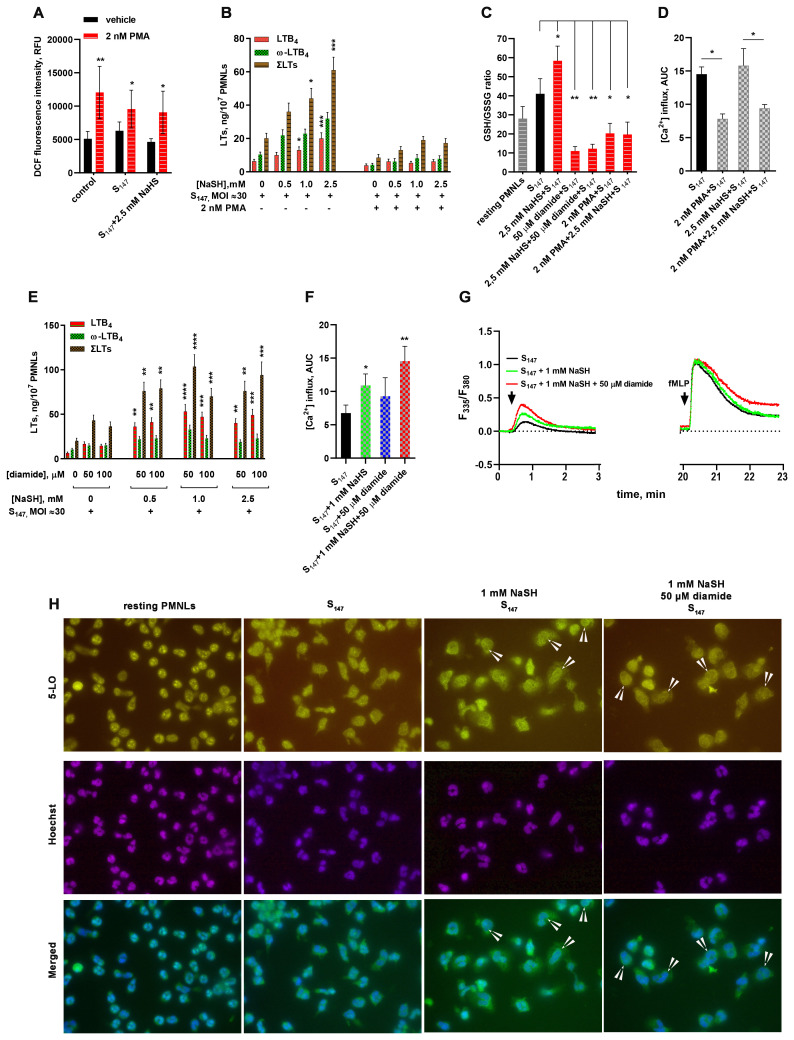
Influence of reactive oxygen species (ROS) and thiol–disulfide oxidative stress on 5-lipoxygenase (5-LOX) activation in neutrophils. **(A)** Phorbol-12-myristate-13-acetate (PMA)-induced accumulation of intracellular ROS. After 10 min pre-incubation, polymorphonuclear leukocytes (PMNLs) loaded with H_2_DCFDA were exposed for 30 min to S_147_ bacteria alone (multiplicity of infection, MOI ≈ 40, with the exception of the controls) or in combination with 2.5 mM sodium hydrosulfide hydrate (NaSH) in the absence (*black*) or the presence (*red*) of PMA. Subsequently, 2',7'-dichlorofluorescein (DCF) fluorescence was measured. Presented are the mean ± SEM of the fluorescence intensity values measured in three independent experiments performed in triplicate. *p < 0.05, **p < 0.01 (compared with the corresponding control values as shown using two-way ANOVA with Tukey’s multiple comparisons test). **(B, E)** Effect of excessive reactive oxygen species (ROS) **(B)** and thiol–disulfide **(E)** oxidative stress on leukotriene synthesis in human neutrophils. PMNLs were exposed for 30 min to S_147_ bacteria alone (bacteria per cell ratio ~30:1) or in combination with NaSH at the indicated concentrations in the absence or presence of PMA **(B)** or diamide **(E)**, followed by the addition of *N*-formyl-l-methionyl-l-leucyl-l-phenylalanine (fMLP, 0.1 µM) for 10 min. After termination of the incubation, the 5-LOX products were analyzed. Presented are the absolute values of LTB_4_ and ω-OH-LTB_4_ and the sum of leukotrienes (LTs) (ΣLTs) in nanograms per 10^7^ PMNLs. Values shown are the mean ± SEM of three independent experiments performed in duplicate. *p<0.05,**p<0.01, ***p<0.001, ****p<0.0001 (for pairs of data compared with the corresponding control values using two-way ANOVA with Tukey’s multiple comparisons test). **(С)** Changes in the glutathione (GSH)/glutathione disulfide (GSSG) ratio under the influence of diamide or PMA-induced oxidative stress. PMNLs were incubated in the presence of S_147_ bacteria (MOI ≈ 40) and the stimuli indicated, except non-treated resting cells, for 20 min, after which the total and oxidized GSH in the cell lysate were determined with a luminescent-based technique. Presented are the mean ± SEM of the GSH/GSSG ratios measured in three independent experiments performed in duplicate. *p < 0.05, **p < 0.01 (for pairs of data indicated as shown by repeated measures (RM) one-way ANOVA with Tukey’s multiple comparisons test). **(D)** Suppression of fMLP-induced Ca^2+^ influx due to ROS hyperproduction. PMNLs loaded with fura-2 AM were exposed for 20 min to S_147_ bacteria alone (MOI ≈ 40) or in combination with 2.5 mM NaSH in the absence or presence of PMA. Subsequently, 0.1 µM fMLP was added with simultaneous fluorescence (335 nm/510 nm and 380 nm/510 nm) detection. Presented are the areas under the curve (AUCs; mean ± SEM) for a 2-min interval after the addition of fMLP to PMNLs. **(F, G)** Enhancement of the bacterium-induced Ca^2+^ influx by NaSH and diamide. PMNLs loaded with fura-2 AM were exposed to S_147_ bacteria alone (MOI = 30–40) or in combination with NaSH and diamide, as indicated. fMLP (0.1 µM) was added 20 min later. Fluorescence was recorded for 3 min after each treatment. Presented are the AUCs (mean ± SEM) for a 2-min interval after the first stimulation. *p < 0.05, **p < 0.01 (compared with the value obtained for the cells treated with S_147_ only, as shown using ordinary one-way ANOVA with Dunnett’s multiple comparisons test) **(F)** and typical kinetic curves (F_380_/F_335_ ratio) for both successive treatments **(G)**. **(H)** Translocation of 5-LOX in cells interacting with bacteria under the influence of NaSH and diamide. PMNLs were incubated for 20 min without stimuli (resting PMNLs) in the presence of S_147_ bacteria alone or with the stimuli indicated followed by co-staining for 5-LOX and double-stranded DNA. Typical images of 5-LOX (*green*), nuclei (*blue*), and their overlays are shown. *Arrows* indicate cells with 5-LOX co-localized with the nuclear membrane.

The addition of nanomolar amounts of phorbol-12-myristate-13-acetate (PMA) to PMNLs at the pre-incubation with bacteria stage caused a moderate increase in the level of intracellular ROS and blocked the synthesis of leukotrienes in response to the addition of fMLP, regardless of the presence of the antioxidant NaSH (**Figures 2A, B**). Under the influence of PMA, a depletion of the intracellular pool of reduced GSH was also observed, which was insensitive to NaSH addition (**Figure 2C**). Moreover, an artificially induced oxidative burst superimposed on the period of PMNL–bacteria interaction suppressed the fMLP-induced calcium influx (**Figure 2D**).

ROS-producing neutrophils efficiently kill bacteria (26, 27). It can be assumed that ROS do not contribute to the increase in leukotriene synthesis in neutrophils as there is no need to attract more neutrophils.

*S. typhimurium* expresses proteins that disrupt the neutrophil NADPH oxidase assembly and alter the ROS production by neutrophils (28). In addition, antioxidant GSH is used by the majority of Gram-negative bacteria, including *Salmonella* (29, 30). Oxidation of GSH and low-molecular-weight (LMW) thiols was observed when *Pseudomonas aeruginosa* is phagocytosed by human neutrophils (31). For protection from irreversible oxidative damage by neutrophil oxidants, bacterial LMW thiols form disulfides with each other and with protein cysteines (so-called S-thiolation) (32, 33). Therefore, oxidative stress can induce the oxidation of thiol groups and the formation of disulfide bonds, supporting easy thiol-based redox switches.

To simulate such a situation, i.e., oxidative stress without ROS, diamide was used (15), under the influence of which a significant decrease in the GSH/GSSG ratio was observed, which was not compensated by the H_2_S donor compound, NaSH (**Figure 2C**). Under diamide-induced disulfide stress, the formation of leukotrienes was slightly increased (**Figure 2E**). At the concentration used, diamide did not exert an independent effect on the accumulation of intracellular ROS and did not suppress the antioxidant effect of NaSH (data not shown). However, in combination with NaSH, it synergistically supported leukotriene synthesis during the interaction of neutrophils with the bacteria *S. typhimurium* (**Figure 2E**).

NaSH and diamide demonstrated the ability to influence the Ca^2+^ influx during the interaction of neutrophils with the bacteria *S. typhimurium* (**Figures 2F, G**). **Figure 2G** presents the Ca^2+^ flux data both during neutrophil–bacteria interaction and at fMLP addition. NaSH and diamide had virtually no effect on fMLP-induced Ca^2+^ response, but caused a significant increase in Ca^2+^ influx when added during PMNL pre-incubation with bacteria (**Figure 2G**). These data are presented in **Figure 2F** as AUCs.

An increase in Ca^2+^ initiates the translocation of 5-LOX into the nuclear membrane (34), which is necessary for 5-LOX activity (35). The stimulation of neutrophils with bacteria, NaSH, and diamide supported the translocation of 5-LOX into the nuclei (**Figure 2H**). In combination with the ROS-producing compound PMA, NaSH did not increase leukotriene synthesis (**Figure 2B**).

In combination with the sulfhydryl (SH)-targeting agent diamide, NaSH increased leukotriene synthesis (**Figure 2E**), i.e., the thiol-mediated signaling events differed from that of ROS-dependent signaling.

### 3.2 H_2_S affects leukotriene synthesis via an energy metabolism

The intracellular ATP concentration is known as an activation factor of 5-LOX (36, 37). Glycolysis plays a major role in ATP production in granulocytes (26, 38). Upon activation, neutrophils switch from glycolysis to the pentose phosphate pathway (PPP) to increase NADPH production; however, this comes at the expense of ATP (39). Stimulation with 10^−7^ M fMLP resulted in the rapid activation of PPP, which was complete at approximately 5 min. When a neutrophil is activated, the hexose monophosphate shunt starts to produce NADPH through the oxidation of glucose-6-phosphate to ribulose-5-phosphate. Oxidative stress addresses more glucose toward the PPP with increased NADPH production.

**Figure 3:**
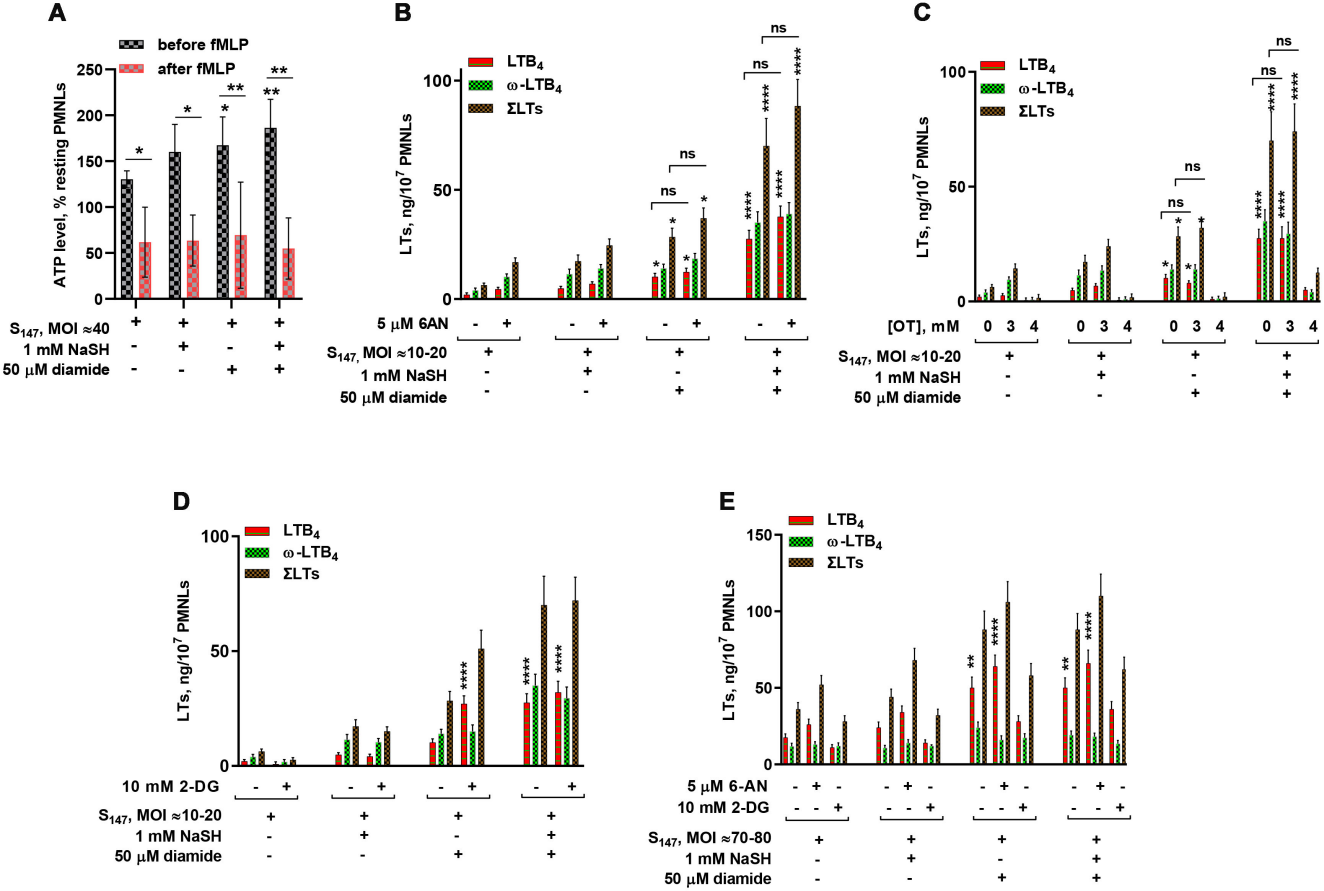
Relationship between leukotriene synthesis and neutrophil energy status. **(A)** Changes in the total ATP levels in neutrophils upon stimulation. Polymorphonuclear leukocytes (PMNLs) were exposed to S_147_ bacteria (multiplicity of infection, MOI ≈ 40) alone or in combination with sodium hydrosulfide hydrate (NaSH) and diamide, as indicated. After 20 min, 0.1 µM *N*-formyl-l-methionyl-l-leucyl-l-phenylalanine (fMLP) was added. Resting cell samples were used as controls for subsequent normalization of the results. After the addition of a pore-forming agent, the total ATP was measured using the bioluminescent method. Presented are the mean ± SEM (*n* = 5) of total ATP as a percentage of the value of resting PMNLs just before (*black*) and 3 min after (*red*) fMLP addition. *p < 0.05, **p < 0.01 [compared with samples treated with S_147_ only or for pairs of data indicated by repeated measures (RM) one-way ANOVA or two-way ANOVA, respectively]. **(B–E)** Effect of 2-deoxy-d-glucose (2-DG), 6-aminonicotinamide (6-AN), and oxythiamine (OT) on leukotriene synthesis in human neutrophils at low/medium (MOI = 10–20) **(B–D)** and high bacterial loads (MOI = 70–80) **(E)**. PMNLs [(1.3–1.6) × 10^7^/6 ml] were pre-incubated for 10 min at 37°C, 5% CO_2_, without or with 5 µM 6-AN, 10 mM 2-DG, and 3 or 4 mM OT, as indicated. After 10 min pre-incubation, the PMNLs were exposed for 30 min to *Salmonella typhimurium* (S_147_) bacteria alone or in combination with 1 mM NaSH and 50 µM diamide, as indicated on the *X*-axis, followed by fMLP (0.1 µM) addition for 10 min. When the incubations stopped, the 5-lipoxygenase (5-LOX) products were analyzed. Values shown are the mean ± SEM of three independent experiments performed in duplicate. *p < 0.05, **p < 0.01, ***p < 0.001, ****p < 0.0001 (for pairs of data compared with the corresponding control values using two-way ANOVA with Tukey’s multiple comparison test).

The neutrophil ATP levels slightly increased after cell incubation with bacteria, but decreased sharply after the addition of fMLP (**Figure 3A**). In experimental conditions corresponding to **Figure 2E**, when NaSH and diamide synergistically increased leukotriene synthesis, the ATP level in neutrophil, detected just before fMLP addition, significantly increased (**Figure 3A**).

H_2_S can modulate the activity of the key enzyme of PPP, glucose-6-phosphate dehydrogenase (G6PD) (40), the enzyme responsible for NADPH accumulation. PPP stimulates NADPH recycling for antioxidant protection. In resting cells, G6PD is inhibited by NADPH (41). The addition of GSH-oxidizing agents such as diamide to HeLa cells resulted in an immediate decrease in the intracellular NADPH dependent on the availability of glucose in the culture medium (42). Increasing both the level of oxidized glutathione (GSSG) and the NADP^+^/NADPH ratio restored the activity of NADPH-inhibited G6PD (43). Indeed, diamide fuels the PPP pathway. Furthermore, diamide in combination with NaSH synergistically increased leukotriene synthesis during the interaction of neutrophils with the bacteria *S. typhimurium* (**Figure 2E**).

Using inhibitors of glycolysis and PPP, we attempted to find functional evidence for the modulation of leukotriene synthesis by the glycolytic and PPP pathways. Without NaSH or diamide, leukotriene synthesis was suppressed by the inhibition of glycolysis with 2-deoxy-d-glucose (2-DG) and was slightly increased by the inhibition of PPP with 6-AN (**Figure 3**).

Diamide is known to stimulate PPP-dependent NADPH production (44). Treatment of cells with diamide (not more than 100 µM) increased protein glutathiolation and influenced cellular bioenergetics, increasing the glycolytic flux (45). This activity can contribute to increased ATP levels in the presence of diamide (**Figure 3A**). Diamide decreased the dependence of leukotriene synthesis on glycolysis inhibition by 2-DG (**Figure 3D**). The slight inhibition of PPP in favor of glycolysis supported leukotriene synthesis (**Figures 3B, E**).

It was reported that H_2_S elevated G6PD activity (40), the key enzyme in PPP. At the same time, NaSH suppressed ROS generation (**Figure 1**), a pathway utilizing NADPH. Reduction in the levels of ROS allows cells to maintain a larger pool of reducing equivalents, in particular NADPH, which eliminates the need for the active turnover of PPP. It was found that, in the presence of NaSH, leukotriene synthesis was not sensitive to the G6PD inhibitor 6-AN and to the inhibitor of glycolysis 2-DG at low bacterial loads (**Figures 3B, D**). At high bacterial loads, the inhibition of glycolysis decreased leukotriene synthesis; however, the inhibition of G6PD with 6-AN increased leukotriene synthesis in the presence of NaSH (**Figure 3E**).

Inhibition of non-oxidative PPP (non-oxPPP) with the transketolase inhibitor OT suppressed leukotriene synthesis at 4 mM, but was not affected at 3 mM (**Figure 3C**). Leukotriene synthesis was sensitive to the inhibition of glycolysis by 2-DG at high bacterial loads (**Figure 3E**). Glycolysis is the main source of ATP in neutrophils (38, 46), and at high bacterial loads, a slight inhibition of oxPPP (in favor of glycolysis) increased leukotriene synthesis (**Figure 3E**).

It can be concluded that redox processes induced by the GSH-supporting compound NaSH and disulfide stress induced by diamide influence the 5-LOX activity also through the energy metabolism in neutrophils.

### 3.3 Effect of H_2_S on ω-hydroxylation of leukotriene B4

LTB_4_ ω-hydroxylation stimulates its transformation into ω-hydroxy-LTB_4_, an endogenous inhibitor of LTB_4_ chemotactic activity (47). When LTB4 is transformed, the signals for attracting neutrophils are attenuated. The increasing concentration gradient of LTB_4_ near the microbial cluster is ensured, among other things, by blocking the ω-OH and ω-COOH transformation of LTB_4_, i.e., self-amplification near the microbial cluster, and a decrease in the concentration of LTB_4_ as the number of bacteria decreases. Such self-reinforcement and self-limitation mechanisms form the self-organized swarming behavior of neutrophils.

**Figure 4:**
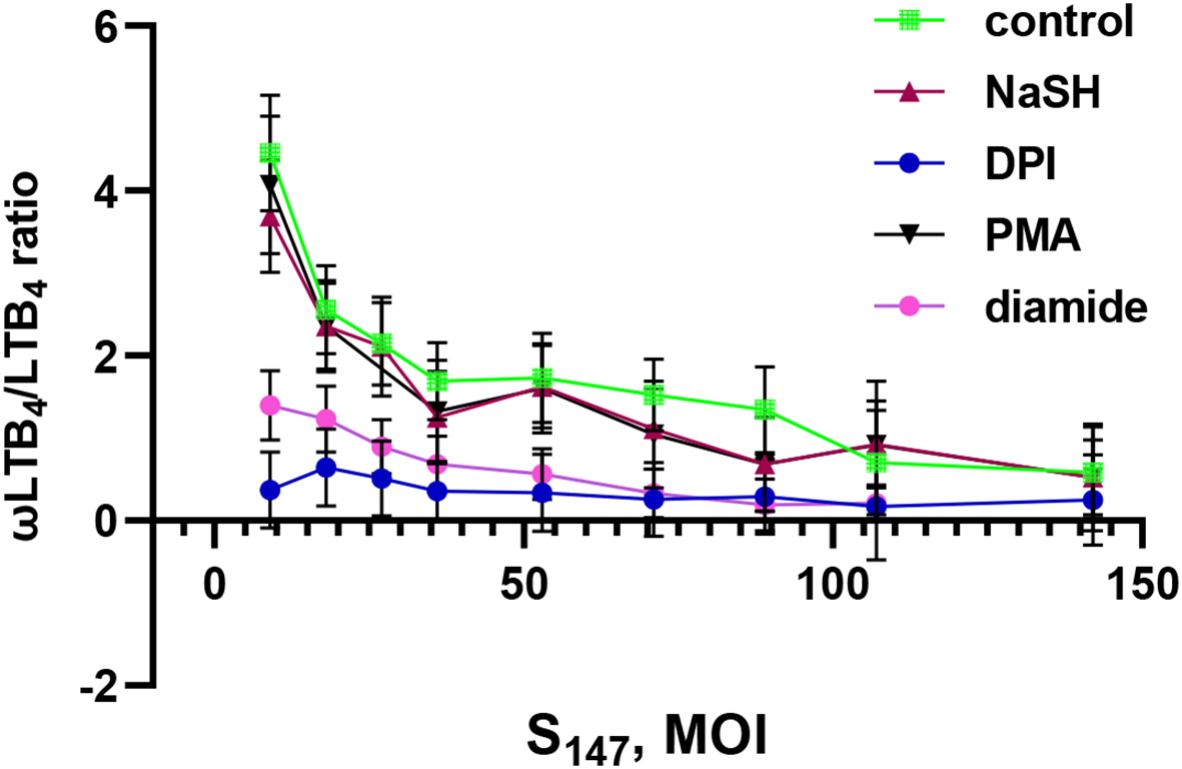
Effect of sodium hydrosulfide hydrate (NaSH), phorbol-12-myristate-13-acetate (PMA), diamide, and diphenyleneiodonium (DPI) on the ω-LTB_4_/LTB_4_ ratio at different bacterial loads. Polymorphonuclear leukocytes (PMNLs) [(1.3–1.6) × 10^7^/6 ml] were pre-incubated for 10 min at 37°C, 5% CO_2_. After 10 min pre-incubation, the PMNLs were exposed for 30 min to *Salmonella typhimurium* (S_147_) bacteria alone or in combination with 1 mM NaSH, 50 µM diamide, 5 µM DPI, and 10 nM PMA, as indicated, followed by *N*-formyl-l-methionyl-l-leucyl-l-phenylalanine (fMLP; 0.1 µM) addition for 10 min. The bacteria-to-PMNL ratio is indicated on the *X*-axis. After termination of the incubation, the 5-lipoxygenase (5-LOX) products were analyzed. Presented are the ω-LTB4/LTB4 ratios. Values shown are the mean ± SEM of three independent experiments performed in duplicate.

Previously, we have shown that LTB_4_ ω-hydroxylation is inhibited at high bacterial loads (19). In this study, it was observed that NaSH supported the ω-hydroxylation of LTB_4_ at low and medium bacterial loads (**Figure 4**). Moreover, it was found that, in the presence of heat-inactivated bacteria, LTB_4_ ω-hydroxylation was not inhibited with increasing bacterial loads (**Supplementary Figure S1**).

The ROS-producing oxidative stress induced by PMA did not change this dependence and supported the ω-hydroxylation of LTB_4_ at low and medium bacterial loads (**Figure 4**). The disulfide stress induced by diamide decreased the ω-hydroxylation of LTB_4_ (**Figure 4**).

When the bacterial load increased, ω-hydroxylation was inhibited in all treatments (**Figure 4**). Interestingly, the same shift in metabolic profile occurred when an NADPH oxidase inhibitor (diphenyleneiodonium, DPI) was added to the cells. The NADPH oxidase inhibitor DPI suppressed the ω-hydroxylation of LTB_4_ regardless of the bacterial load (**Figure 4**). This indicates that self-limitation of swarming, based on the ω-hydroxylation of LTB_4_, will not work during this treatment. A recently published paper has shown that neutrophils from patients with chronic granulomatous disease (i.e., with dysfunctional NADPH oxidase) formed a swarm that grew continuously and disproportionately to the point of invasion (48).

Neutrophil swarming is controlled by LTB_4_ and its interaction with its receptor, BLT1 (49). The disulfide stress induced by diamide, in combination with NaSH, resulted in a significant enhancement in LTB_4_ synthesis, in parallel with the decreased ω-hydroxylation of LTB_4_ (**Figure 2**), which may accelerate the accumulation of neutrophils and the formation of clusters around the invading microorganism.

## 4 Discussion

Neutrophils fighting against bacteria communicate with each other, and for this communication, they use LTB_4_. Neutrophils can produce LTB_4_ and can respond to LTB_4_ when this molecule binds to the LTB_4_ receptor 1 (BLT1) on neutrophils. The synthesis of LTB_4_ is very important for the initiation of swarming to the microbial cluster (50). How do neutrophils distinguish between the need and the lack of need to recruit more neutrophils?

Using the type III secretion system (T3SS), *Salmonella* manipulates host processes (51), including the host lipid metabolism (52). T3SS and flagellar motility are potent factors in *S. typhimurium*-induced neutrophil respiratory burst (53). These activities depend on the bacterial viability. *Salmonella* activates the host PLA2 activity (54, 55). The pathogenic bacteria *Yersinia* suppressed the Ca^2+^ response in human neutrophils (56).

Neutrophils play an important role in the host defense against *Salmonella* infection and are key cells involved in the dissemination of *S. typhimurium* (57). Non-typhoidal *Salmonella* responding to oxidative stress produce H_2_S (9). H_2_S is used by bacteria as a universal protective reagent against host cells and is a vital factor in the formation of bacterial biofilms. H_2_S scavengers enhance the clearance of intracellular bacteria in neutrophils and macrophages (58).

The plasma H_2_S concentration increased in lipopolysaccharide (LPS)-induced inflammation (59). Protein *S*-sulfhydration may be a possible effect of H_2_S (60). H_2_S controls the cellular Ca^2+^ level through Ca^2+^ channel sulfhydration, which influences cell signaling (25). H_2_S also induces the *S*-sulfhydration of many proteins, including potassium cannels (61), endothelial nitric oxide synthase (eNOS) (62), and MEK1 (63).

It is a well-known fact that the leading microbicidal mechanism is the ability of phagocytic cells to produce large amounts of oxidants. In the course of evolution, pathogenic microorganisms have developed a number of mechanisms that allow them not only to survive but also to use ROS-dependent mechanisms of the immune response to their advantage. It has been shown that *Salmonella enterica* virulence depends primarily on the ability to induce overwhelming systemic oxidative stress, mediated by bacterial thioredoxin 1 (64, 65). The latter is, among other things, a component of enzymatic cascades leading to the formation of hydrosulfide (66). In the presence of live bacteria producing H_2_S, the formation of ROS is suppressed, and in this connection, it was interesting to compare the neutrophil responses to ROS- and disulfide-induced oxidative stress.

An unexpected finding in this study is that of a fundamental difference in the regulation of leukotriene synthesis in response to ROS- or disulfide-induced oxidative stress. Thiol–disulfide homeostasis stabilizes the protein structures and regulates the functions of proteins, receptors, and ion channels. Oxidative stress affects thiol–disulfide homeostasis, and 5-LOX and leukotriene synthesis appear to be very sensitive to these fluctuations.

Targeted at the thiol–disulfide homeostasis, oxidative stress can be induced by chemical agents penetrating into cells. Diamide is a cell-penetrating oxidant that specifically targets GSH thiols and free SH groups of proteins (15). Diamide is an oxidizing agent of intracellular thiols and increases the GSSG-to-GSH ratio (67). It may inhibit protein tyrosine phosphatases (68), which could result in increased phosphorylation of tyrosine kinases p38 MAPK (69) and ERK (70).

The combination of the H_2_S donor NaSH with the thiol-oxidizing agent diamide produced a strong stimulating effect on leukotriene synthesis. The cellular peroxide status is very important for 5-LOX activation (6, 7). Oxidative ROS-mediated processes induced by PMA during the incubation of neutrophil with bacteria in the absence of the end-target chemoattractant fMLP further suppressed the 5-LOX activation upon fMLP addition (**Figure 2**). In contrast, the diamide-induced disulfide stress did not stimulate ROS production, but facilitated increased fatty acid hydroperoxide formation (6). This can facilitate the onset of lipoxygenase activity (71) and in parallel with the increased Ca^2+^ induced by fMLP collectively support 5-LOX activity.

The activity of 5-LOX requires intact energy metabolism, and the activity drops with the decrease of intracellular ATP (72). Glycolysis produces the most energy, at a rate of two ATPs and two pyruvate molecules per glucose, but without NADPH. A rapid transition to the pentose cycle is required to enhance the oxidative burst and the associated effector functions in activated neutrophils, ultimately allowing them to rapidly establish a first line of defense against pathogens. PPP can be transiently activated in response to oxidative stress or during the oxidative burst of phagocytes to meet the urgent need for NADPH (73). Antioxidant systems including GSH and thioredoxin use NADPH to regenerate reduced thiols from disulfides. NaSH increases the ability of fMLP/diamide to activate the cells. Reduction in the levels of ROS allows cells to maintain a larger pool of reducing equivalents, in particular NADPH, thereby increasing the cell resources and providing greater activation. In the presence of NaSH, the cells were quite tolerant to the addition of PPP inhibitors (**Figure 3**). Diamide is known to stimulate PPP-dependent NADPH production (44), but not at the expense of ATP; instead, it provides prolonged increase in glycolytic flux into cells (45).

To summarize, our research showed that the disruption of the thiol–disulfide balance plays a decisive role in the synthesis of LTB_4_, ensuring neutrophil swarming to microbial clusters. The induction of this redox imbalance may initiate a cascade of molecular signaling to 5-LOX activation and LTB_4_ formation. Of fundamental importance is the suppression of ROS signaling, which is associated with additional production of NADPH, at the cost of a lower ATP production (39). However, disruption of the thiol–disulfide status is perceived by neutrophils as a distress signal in the fight against microbes.

## Data Availability

The raw data supporting the conclusions of this article will be made available by the authors, without undue reservation.
