# Supplementary material for: Sulfur compounds navigate redox processes, leukotriene synthesis, and ω-hydroxylation of leukotriene B4 in neutrophil interaction with the bacteria Salmonella typhimurium: the way to manipulate neutrophil swarming
Source: Front Immunol. 2025 Oct 15;16:1606408. doi: 10.3389/fimmu.2025.1606408 (PMC12568030; doi:10.3389/fimmu.2025.1606408)
Supplement: Supplementary file 1 [file DataSheet1.pdf]

Supplementary Material

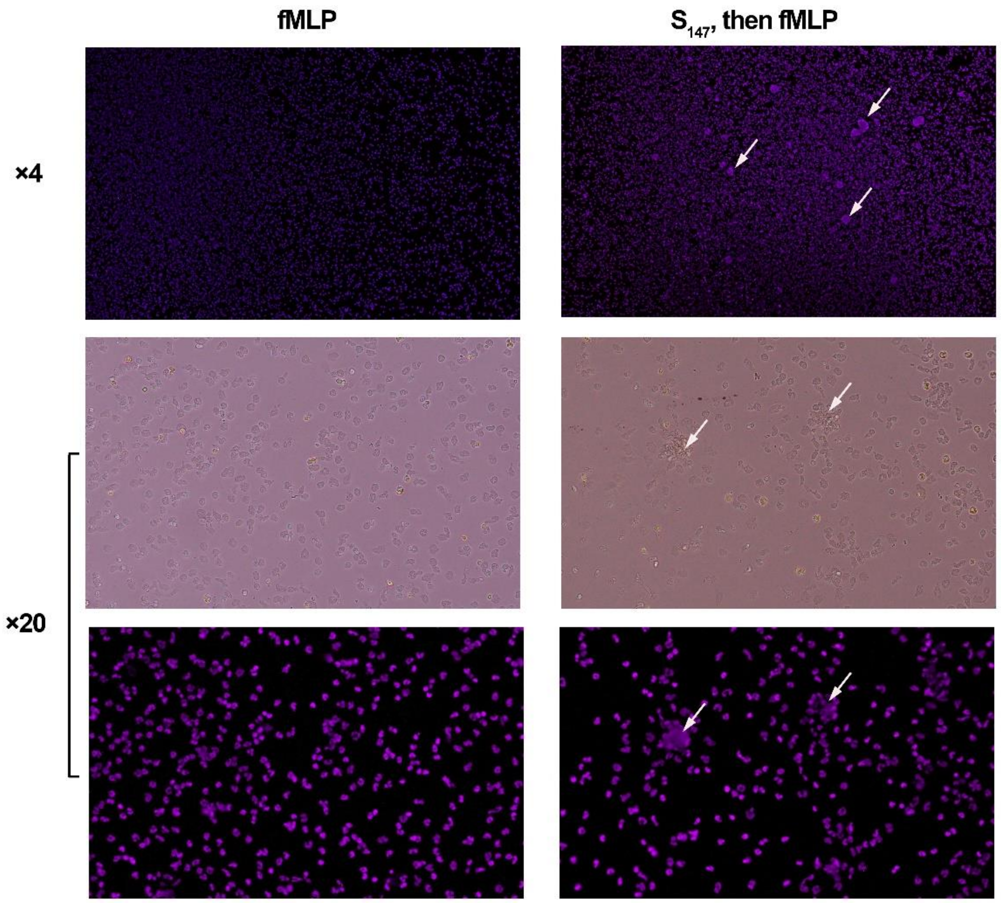

Supplementary Figure S1. Formation of local neutrophil clasters (white arrows) under conditions of sequential exposure to bacteria and fMLP. PMNs (10^6/mL HBSS/HEPES) were incubated in the absence (left) or presence (right) of Salmonella typhimurium (S_{147}) bacteria (MOI ≈40) for 20 min (glass bottom dishes, 37 °C, 5% CO_2). Then 0.1 µM fMLP was added for 5 min followed by fixation with 2.5% PFA and staining with 2 µM Hoechst 33342. Image acquisition was performed using a fluorescence microscope Olympus IX 83 (Japan). At least 8 random pictures were captured for each sample.

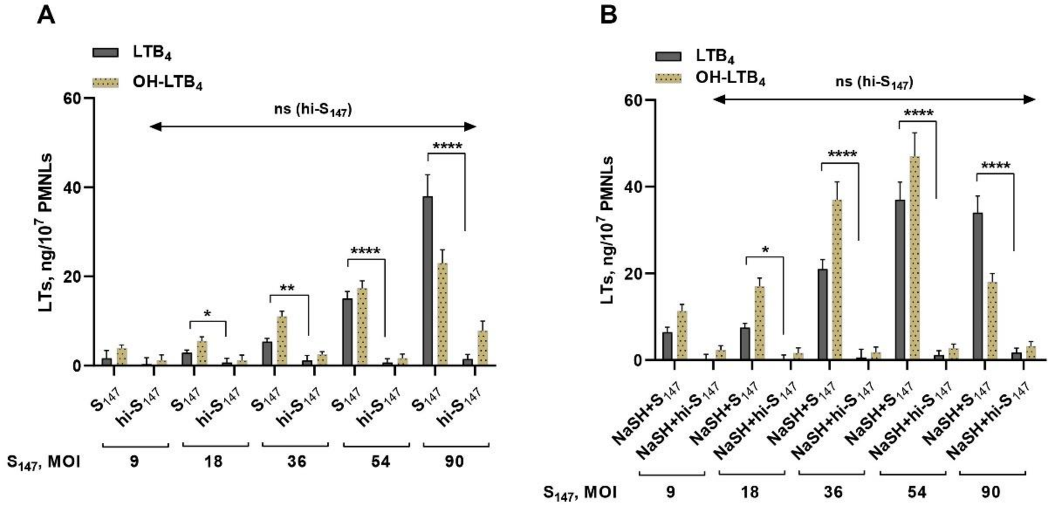

Supplementary Figure S2. Leukotriene synthesis in human neutrophils at various bacterial loads. PMNs (1.3-1.6) x10^7/6 ml were pre-incubated for 10 min at 37 ºC, 5% CO_2. Human neutrophils were exposed to *Salmonella typhimurium* (S_{147}) for 30 min, without (A) or with 1mM NaSH, followed by fMLP (0.1 µM) addition for 10 min; the ratio of bacteria to PMNLs (MOI) is indicated. Hi-S_{147} stands for heat inactivated bacteria. The 5-LOX products were analyzed using HPLC, and data for LTB_4 and ω-OH-LTB_4 are presented. Values indicate mean ± SEM of three independent experiments performed in duplicate. *$p < 0.05$, **$p < 0.01$, ****$p < 0.0001$, for pairs of data compared to corresponding control values by two-way ANOVA with Tukey's multiple comparison test.

The data show that the effect of heat-inactivated bacteria was much smaller than that of live bacteria and did not change with increasing bacterial load.

.
